# Combined Use of Vitamin D and DPP-4 Inhibitors as a Potential Adjuvant Treatment Strategy to Enhance the Efficacy of Novel Beta-Cell Replacement Therapies for Type 1 Diabetes

**DOI:** 10.3390/medsci13030141

**Published:** 2025-08-18

**Authors:** Marcelo Maia Pinheiro, Felipe Moura Maia Pinheiro, Bruna Fioravante Di Serio, Nathalia Padilla, Benjamin Udoka Nwosu, David Della-Morte, Camillo Ricordi, Marco Infante

**Affiliations:** 1 Faculty of Medicine, Centro Universitário de Várzea Grande, UNIVAG, Av. Dom Orlando Chaves 2655-Cristo Rei, Várzea Grande 78118-000, Mato Grosso, Brazil; 2 Faculty of Medicine, UNIC—Universidade de Cuiabá, Av. Manoel José de Arruda 3100-Jardim Europa, Cuiabá 78065-900, Mato Grosso, Brazil; felpsmoura@gmail.com; 3 Beta Cell Center, R. das Papoulas 332, Cuiabá 78043-138, Mato Grosso, Brazil; bfdiserio@hotmail.com; 4 Division of Cellular Transplantation, Department of Surgery, Cell Transplant Center, Diabetes Research Institute (DRI), University of Miami Miller School of Medicine, 1450 NW 10th Ave., Miami, FL 33136, USA; nathalia.padilla22@gmail.com (N.P.); cricordi@med.miami.edu (C.R.); or marco.infante@unicamillus.org (M.I.); 5 Division of Endocrinology, Department of Pediatrics, Zucker School of Medicine at Hofstra/Northwell, 500 Hofstra Blvd, Hempstead, NY 11549, USA; bnwosu1@northwell.edu; 6 Section of Clinical Nutrition and Nutrigenomics, Department of Biomedicine and Prevention, University of Rome Tor Vergata, Via Montpellier 1, 00133 Rome, Italy; david.dellamorte@uniroma2.it; 7 Department of Neurology, Evelyn F. McKnight Brain Institute, University of Miami Miller School of Medicine, 1120 NW 14th Street, Miami, FL 33136, USA; 8 Section of Diabetes & Metabolic Disorders, UniCamillus—Saint Camillus International University of Health Sciences, Via di Sant’Alessandro 8, 00131 Rome, Italy

**Keywords:** vitamin D, CD26, dipeptidyl peptidase-4, DPP-4 inhibitors, VIDPP-4i, islet transplantation, beta-cell replacement, islet encapsulation, type 1 diabetes, T1D

## Abstract

Emerging evidence suggests that vitamin D and dipeptidyl peptidase-4 (DPP-4) inhibitors exert synergistic immunomodulatory, anti-inflammatory and antioxidant actions. Moreover, intervention studies showed that combination therapy based on the concomitant use of vitamin D and DPP-4 inhibitors (VIDPP-4i) may preserve beta-cell function in patients with type 1 diabetes mellitus (T1D) and latent autoimmune diabetes in adults (LADA). These effects are particularly relevant in the context of beta-cell replacement strategies, whose long-term efficacy can be hampered by various factors, such as immune-mediated graft rejection, inadequate vascularization, hypoxia, trauma-induced cell apoptosis, fibrosis, host immune response, and recurrence of autoimmunity. Based on preclinical and clinical studies conducted in the fields of autoimmune diabetes and solid organ/cell transplantation, the present narrative review aims to describe the rationale behind the investigation of VIDPP-4i combination therapy as an adjuvant treatment strategy to enhance the efficacy of novel beta-cell replacement therapies for T1D. In this regard, we discuss the potential immune and metabolic mechanisms through which vitamin D and DPP-4 inhibitors can promote the long-term function and survival of transplanted islets in patients with T1D receiving various types of beta-cell replacement therapies, including therapeutic approaches using encapsulated stem cell-derived beta cells.

## 1. Introduction: Beta-Cell Replacement Therapies for Type 1 Diabetes

Type 1 diabetes mellitus (T1D or T1DM) is an organ-specific autoimmune disease characterized by the gradual, immune-mediated destruction of insulin-secreting pancreatic beta cells, which ultimately leads to lifelong dependence on exogenous insulin [1]. Currently, there is still no definitive biological cure for T1D. Nevertheless, after a century of almost exclusive reliance on insulin therapy for the treatment of T1D, novel immunotherapies are now focused on immunomodulation for patients with pre-symptomatic disease (stages 1 and 2 T1D) and for patients with newly diagnosed (symptomatic) disease (stage 3 T1D) [2]. On the other hand, novel beta-cell replacement strategies aim at restoring pancreatic beta-cell mass and function in patients with new-onset and established T1D [3]. Islet transplantation has proven effective in restoring endogenous insulin secretion and hypoglycemia awareness in T1D patients [4]. Yet, organ donor shortage, risk of graft rejection, and the need for chronic immunosuppression restrict the adoption of islet transplantation to a limited subset of patients with T1D [5,6]. Another major challenge in clinical islet transplantation is the considerable islet cell loss occurring early and late following intrahepatic islet transplantation due to various factors, such as the instant blood-mediated inflammatory reaction (IBMIR), pronounced host autoimmune and alloimmune responses, as well as beta-cell toxicity induced by immunosuppressants [7]. Therefore, different strategies are under study to circumvent these detrimental events, such as the investigation of alternative transplantation sites, as well as the transplantation of stem cell-derived insulin-producing beta cells [3,7].

Over the past few years, remarkable advancements in stem cell biology, tissue engineering, nanotechnology, and gene editing have driven a paradigm shift in the field of beta-cell replacement therapies for T1D [5,8]. The biotechnology company ViaCyte—acquired by Vertex Pharmaceuticals in 2022—has contributed to the development of the investigational stem cell-derived, fully differentiated pancreatic islet cell therapies Zimislecel (formerly known as VX-880) and VX-264. Zimislecel is an investigational allogeneic stem cell-derived, fully differentiated, insulin-secreting islet cell therapy, which is delivered through an infusion into the hepatic portal vein and requires the chronic use of immunosuppressants to prevent the immune-mediated rejection of islet cells [9,10]. The recently published phase 1–2 VX-880-101 FORWARD study investigated the safety and efficacy profile of Zimislecel in adults with T1D who had impaired awareness of hypoglycemia and experienced repeated episodes of severe hypoglycemia [11]. Authors assessed Zimislecel at a half dose (0.4 × 10^9^ cells) in part A of the study and at a full dose (0.8 × 10^9^ cells) in parts B and C of the study. Zimislecel was administered through a single infusion into the portal vein over a period of 30–60 min. Study participants had a mean duration of diabetes of 22.8 years. Furthermore, all study participants received glucocorticoid-free immunosuppressive therapy. Overall, 14 participants (2 participants in part A; 12 participants in parts B and C) were included in the analyses, as they completed at least 12 months of follow-up. At baseline, fasting C-peptide (a biomarker of endogenous insulin secretion) was not detectable in all study participants. All participants exhibited engraftment and islet function after Zimislecel infusion, as documented by detectable fasting and mixed-meal tolerance test (MMTT)-stimulated serum C-peptide levels. Remarkably, 10 of the 12 participants (83%) in parts B and C of the study exhibited insulin independence at day 365. Moreover, all 12 participants enrolled in parts B and C of the study remained free of severe hypoglycemic episodes, showed a glycated hemoglobin (HbA1c) value lower than 7%, and exhibited more than 70% of the time spent in the target glucose range (70–180 mg/dL) at day 365. No serious adverse events were considered by investigators to be related (or possibly related) to Zimislecel [11].

On the other hand, VX-264 is an investigational cell therapy in which allogeneic human stem cell-derived islets are encapsulated in a channel array device designed to shield the islet cells from the attack mediated by the immune system [12]. However, Vertex recently announced that the phase 1/2 VX-264 study (based on the investigation of the fully differentiated pancreatic islet cell therapy encapsulated within a proprietary immunoprotective device) did not meet the efficacy endpoint [10]. VX-264 was generally safe and well-tolerated, but the increases in stimulated C-peptide were not observed at levels required to provide benefit [10]. Thus, VX-264, which was originally designed to be surgically implanted without the need for immunosuppression to protect the islet cells [12], will not be advancing further in clinical trials [10].

A major limitation of the current encapsulation devices containing stem cell-derived pancreatic beta cells is the partial post-transplant loss of beta cells caused by various factors, such as inadequate vascularization, hypoxia, trauma-induced cell apoptosis, fibrosis, and host immune response, which can reduce the long-term efficacy of such therapeutic strategies [13]. In particular, inadequate vascularization represents a major challenge in islet encapsulation, with functional vasculature around an islet implant ensuring proper oxygen and nutrient supply, removal of metabolic waste, and rapid insulin release kinetics [14]. Hence, there is a strong demand for novel approaches aimed at promoting and maintaining proper vascularization of the islet cell implants, ensuring adequate supply of oxygen and nutrients to beta cells, and preventing the dysfunction of the transplanted islets. Moreover, it should be highlighted that T1D is often accompanied by many abnormalities other than the loss and dysfunction of beta cells, including the dysfunction of glucagon-secreting pancreatic alpha cells [15], histological abnormalities of the exocrine pancreas [16], as well as increased serum activity of the enzyme dipeptidyl peptidase-4 (DPP-4) [17], among others.

In light of the aforementioned considerations, safe and cost-effective approaches to mitigate the risk of failure of beta-cell replacement strategies are highly required. Based on preclinical and clinical studies conducted in the field of autoimmune diseases and solid organ and cell transplantation, the present narrative review aims to describe the rationale behind the investigation of combination therapy with vitamin D plus DPP-4 inhibitors (DPP-4i) [18] as a potential adjuvant treatment strategy to enhance the efficacy of novel beta-cell replacement therapies for T1D.

## 2. Impact of Vitamin D on Inflammation, Autoimmunity, and Type 1 Diabetes

Vitamin D has been shown to exert immunomodulatory and anti-inflammatory actions besides its known role in the regulation of calcium-phosphorus homeostasis and bone metabolism [19]. Remarkably, vitamin D receptor (VDR) has been detected in distinct immune cells, such as monocytes, professional antigen-presenting cells (APCs: dendritic cells, macrophages, B cells), T cells, neutrophils, and microglia [20,21,22,23,24,25,26,27]. Moreover, both murine and human APCs express 25-hydroxyvitamin D(3)-1α-hydroxylase [28,29], which is the enzyme responsible for the conversion of 25-hydroxyvitamin D3 (a.k.a. calcifediol) into the biologically active form of vitamin D3 called 1,25-dihydroxyvitamin D3 (a.k.a. calcitriol) [19].

Besides serving as vitamin D targets, immune cells also act as local sources of vitamin D production [30]. It has been shown that various immune cells—such as monocytes, macrophages, dendritic cells, T cells, and B cells—express vitamin D-activating enzymes [20,28,29,31,32,33]. Moreover, it has been documented that calcitriol inhibits the production of pro-inflammatory cytokines by monocytes and macrophages [34], reduces the T-cell stimulatory capacity of macrophages [35], stimulates the transition of macrophages from a pro-inflammatory state (M1 or “classically activated” macrophages) to an anti-inflammatory state (M2 or “alternatively activated” macrophages) [36], renders the dendritic cells more tolerogenic [37], promotes the development of regulatory T cells (Tregs) [38], and favors the transition of T cells from an “effector” phenotype to a “regulatory” (anti-inflammatory) phenotype by enhancing the T-helper (Th) 2 cell development and decreasing the differentiation of Th1 and Th17 cells [39,40,41]. Moreover, a systematic review and meta-analysis of clinical trials found that vitamin D supplementation has the potential to exert antioxidant actions [42].

Additionally, preclinical evidence indicates that vitamin D can regulate insulin synthesis and secretion from pancreatic beta cells [43]. Human pancreatic beta cells express both 25-hydroxyvitamin D(3)-1α-hydroxylase [44,45] and VDR [46]. Moreover, a vitamin D response element (VDRE) has been detected in the human insulin receptor gene promoter [47]. Wei et al. [48] described a VDR-dependent transcriptional program sustaining beta-cell survival through anti-inflammatory responses. Morró et al. [49] demonstrated that transgenic mice with VDR overexpression in pancreatic beta cells are protected against the development of streptozotocin-induced diabetes and show reduced islet inflammation and preserved beta-cell mass. In addition, evidence suggests that vitamin D may promote insulin secretion and enhance insulin sensitivity. In fact, mice lacking a functional VDR show an altered insulin secretory capacity [43]. Bourlon et al. [50] found that calcitriol stimulates insulin biosynthesis and promotes the conversion of proinsulin to insulin in the rat pancreatic islets. Accordingly, calcitriol administration has been shown to increase insulin secretion in vitamin D-deficient rats [51]. Some observational studies documented an inverse correlation between serum 25-hydroxyvitamin D [25(OH)D] levels and insulin resistance [52,53], although this finding was not confirmed by other studies [54,55]. Indeed, a study conducted on subjects with overweight/obesity found that neither plasma 25(OH)D3 concentrations nor plasma 1,25-dihydroxyvitamin D3 concentrations were associated with hepatic, adipose tissue, and peripheral insulin sensitivity [55]. Interestingly, a 6-month randomized, placebo-controlled trial demonstrated that high-dose vitamin D3 supplementation (5000 IU/day) significantly increased peripheral insulin sensitivity and beta-cell function in individuals with newly diagnosed type 2 diabetes (T2D) or at high risk of diabetes [56].

Hypovitaminosis D has been increasingly recognized as a risk factor for various autoimmune diseases, including T1D [19,57,58]. Studies suggested that vitamin D intake and higher serum vitamin D levels during infancy and early childhood may decrease the risk of T1D development later in life [58,59,60]. Hence, vitamin D supplementation has been suggested as a valid tool for the prevention and treatment of different autoimmune diseases, including T1D [57,58,61,62].

Numerous preclinical studies involving non-obese diabetic (NOD) mice—a well-established animal model of human T1D [63]—documented that calcitriol and its analogs can prevent the development or counteract the progression of insulitis and autoimmune diabetes [64,65,66,67,68]. Several observational studies demonstrated that patients with new-onset and long-standing T1D exhibit significantly lower serum vitamin D levels as compared to healthy controls [69,70,71,72,73,74,75,76,77,78]. Randomized controlled trials conducted in patients with new-onset T1D showed that the administration of cholecalciferol (a.k.a. vitamin D3) increased the percentage and the suppressive capacity of Tregs [79,80]. Recently, a post hoc secondary analysis of the POSEIDON trial documented that serum 25(OH)D levels are directly associated with fasting serum C-peptide in youth and adults with recent-onset T1D [81]. This study also documented that fasting serum C-peptide levels were significantly lower in subjects with hypovitaminosis D than in subjects with sufficient serum 25(OH)D levels [81]. These findings suggested that low serum 25(OH)D levels may be associated with more aggressive beta-cell autoimmunity and lower preservation of beta-cell mass and function in subjects with recent-onset T1D. Another study documented that the administration of calcifediol for 12 months—designed to attain serum 25(OH)D values greater than 50 ng/mL—was associated with reduced peripheral blood mononuclear cell reactivity against proinsulin and glutamic acid decarboxylase 65 (GAD65) upon 25(OH)D3 replenishment, along with stable fasting C-peptide levels [82]. In keeping with these findings, we previously described the case of a 22-year-old man with recent-onset T1D who received calcifediol soon after the disease diagnosis and exhibited a prolonged duration (31 months) of the clinical remission phase of T1D [83]. A 12-month randomized controlled trial conducted in youth with new-onset T1D demonstrated that vitamin D2 (a.k.a. ergocalciferol; dose: 50,000 IU/week for 2 months, and then once every 2 weeks for 10 months) significantly reduced serum tumor necrosis factor (TNF)-alpha concentration and the rates of increase in HbA1c and insulin dose-adjusted HbA1c (IDAA1c) [84]. These findings suggested that ergocalciferol (a.k.a. vitamin D2) may preserve residual beta-cell function and sustain partial clinical remission of T1D. A post hoc secondary analysis of the aforementioned trial [85] found that vitamin D2 significantly decreased fasting proinsulin-to-C-peptide ratio—a biomarker of beta-cell endoplasmic reticulum stress [86]—and slowed the decrease in the area under the curve (AUC) of C-peptide in youth with newly diagnosed T1D [85]. Moreover, it is important to highlight that vitamin D supplementation has proven beneficial in autoimmune diseases other than T1D. Recently, a parallel, double-blind, randomized placebo-controlled clinical trial demonstrated that 24-month high-dose vitamin D3 administration (at a dose of 100,000 IU every 2 weeks) significantly decreased disease activity in adults with clinically isolated syndrome and early relapsing-remitting multiple sclerosis [87].

Vitamin D also appears to be involved in wound healing. In mice with streptozotocin-induced diabetes mellitus, 1,25-dihydroxyvitamin D3 has been shown to accelerate diabetic wound healing by alleviating inflammation, improving vascular endothelial dysfunction, and stimulating angiogenesis via the upregulated expression of angiogenic factors, such as Vascular Endothelial Growth Factor (VEGF) [88]. Furthermore, 1,25-dihydroxyvitamin D3 has been shown to accelerate wound healing by suppressing endoplasmic reticulum stress in mice with streptozotocin-induced diabetes mellitus [89]. Notably, a study conducted on ex vivo human skin explants and primary cell culture models documented that 1,25-dihydroxyvitamin D3 increases early wound closure rate by accelerating keratinocyte migration and promoting re-epithelialization, and reduces extracellular matrix remodeling by inhibiting fibroblast migration and fibroblast transition into profibrotic myofibroblasts [90]. These findings indicate that optimizing vitamin D status may favor wound healing while minimizing excessive scarring and fibrosis.

A recent meta-analysis of randomized controlled trials evaluating the impact of vitamin D on diabetic foot ulcers showed that vitamin D supplementation can promote diabetic foot ulcer healing by improving glucose control and attenuating oxidative stress and inflammation [91]. Notably, a randomized, double-blind clinical trial recently showed that the improvement in the healing of diabetic foot ulcers occurred particularly when a high cholecalciferol dose (amounting to a daily oral intake of 170 μg) was used [92].

## 3. Role of Vitamin D in Solid Organ and Cell Transplantation

Preclinical studies conducted in animal models of allogeneic and syngeneic islet transplantation showed that vitamin D and its analogs promote islet graft survival and prevent or delay recurrence of autoimmunity and allograft rejection [93,94,95,96,97,98,99]. Furthermore, observational studies documented that low serum vitamin D levels can worsen clinical outcomes in patients undergoing solid organ transplantation. Fotros et al. [100] showed that vitamin D deficiency during the pre-transplant period is significantly associated with an increased risk of acute cellular rejection in patients with cirrhosis undergoing liver transplantation. In a retrospective study conducted on lung transplant recipients, Ki et al. [101] found that subjects with vitamin D deficiency exhibit a greater incidence of post-transplant pneumonia and overall mortality as compared to subjects with sufficient serum vitamin D levels. Koimtzis et al. [102] documented that vitamin D deficiency is associated with adverse short-term and long-term outcomes after kidney transplantation, including greater incidence of acute rejection episodes, worse graft function, greater incidence of proteinuria, viral infections, and lower overall graft and patient survival rates. Additionally, vitamin D deficiency is highly prevalent in patients undergoing hematopoietic stem cell transplantation and may influence the risk of developing chronic graft-versus-host disease (GVHD) in this population [103].

## 4. Impact of DPP-4/CD26 and DPP-4 Inhibitors on Inflammation, Autoimmunity, and Type 1 Diabetes

The enzyme DPP-4 (a.k.a. CD26 or cluster of differentiation 26) is a multifunctional cell surface antigen expressed in various cells and tissues, including endothelia, kidney, lung, liver, intestine, pancreatic duct and islet cells, fibroblasts, and immune cells, such as dendritic cells, monocytes, macrophages, activated B cells, T cells, and activated natural killer (NK) cells [104,105,106]. Besides its enzymatic activity, DPP-4 serves as a binding protein and as a ligand for various extracellular molecules [107]. DPP-4 is a type II transmembrane, homodimeric glycoprotein anchored to the cell membrane by its signal peptide [104], although it also exists in a soluble circulating form, which is released from the cell membrane into the bloodstream and accounts for a significant proportion of DPP-4 activity in the human serum [104,108]. DPP-4 mediates the cleavage and inactivation of the insulinotropic (gut-derived) hormones GLP-1 (Glucagon-like peptide 1) and GIP (Glucose-dependent insulinotropic polypeptide), which are also known as “incretins” [109,110]. DPP-4/CD26 also acts as a potent co-stimulatory factor involved in T-cell activation and proliferation [111]. Moreover, approximately 50% of human B lymphocytes express DPP-4/CD26 upon their activation [104,112].

DPP-4 inhibitors (DPP-4i) are oral glucose-lowering medications approved for the treatment of T2D due to their ability to improve glucose control by extending the half-life and biological activity of endogenous GLP-1 and GIP [113,114]. However, evidence suggests that DPP-4i exert pleiotropic effects beyond their glucose-lowering actions. Notably, studies suggested that DPP-4i exert in vitro and in vivo anti-inflammatory and immunomodulatory actions [115,116,117,118,119,120,121], which could be leveraged for the treatment of various chronic inflammatory and autoimmune diseases. In this regard, we previously documented that the DPP-4 inhibitor sitagliptin inhibits human peripheral blood mononuclear cell proliferation in a dose-dependent manner and reduces Th1/Th17 cell differentiation in vitro [122]. A study conducted on human isolated pancreatic islets demonstrated that the DPP-4 inhibitor linagliptin improves beta-cell function and survival, reduces oxidative stress, and counteracts glucotoxicity, lipotoxicity and cytokine-induced toxicity [118].

Additional evidence suggests that DPP-4/CD26 plays a role in the pathogenesis of various chronic fibrotic diseases, such as lung fibrosis, liver cirrhosis, cardiac fibrosis, kidney fibrosis, and systemic sclerosis [123]. Indeed, the inhibition of DPP-4/CD26 has been shown to reduce fibrotic changes and to modulate the profibrotic tissue microenvironment within affected organs in chronic fibrotic diseases [123]. Interestingly, it has been shown that DPP-4/CD26 affects periwound inflammation, extracellular matrix secretion, re-epithelialization, and skin fibrosis [124]. Selective inhibition of fibroblast- and keratinocyte-derived DPP-4/CD26 has been proposed as a therapeutic tool to inhibit skin fibrosis and prevent proliferative scarring and keloid scar formation [124]. In this regard, preclinical studies have shown that DPP-4i improve wound healing and reduce scar formation by promoting angiogenesis, epithelialization, epithelial-mesenchymal transition, and bone marrow-derived mesenchymal progenitor cell population recruitment to wounds [125,126,127,128].

Marfella et al. [129] showed that the DPP-4 inhibitor vildagliptin (administered at the dose of 50 mg twice daily) may accelerate the healing of chronic foot ulcers in patients with T2D by enhancing angiogenesis (as evidenced by increased ulcer capillary density and VEGF expression levels) and reducing the levels of nitrotyrosine (a marker of oxidative stress) within the ulcer specimens. Accordingly, a randomized, double-blind, placebo-controlled trial conducted on 50 adults with T2D and diabetic foot ulcers showed that 12-week vildagliptin therapy (at a daily dose of 100 mg)—in addition to standard of care for diabetic foot ulcers—led to an estimated 35% increase in diabetic foot ulcer healing capacity as compared to placebo combined with the standard of care for diabetic foot ulcers [130]. Furthermore, DPP-4i may exert proangiogenic actions in pancreatic islets. Indeed, in a study conducted on diabetic mice undergoing the implantation of mouse or porcine donor islets under the kidney capsule, the use of sitagliptin increased the mean insulin content of islet grafts and beta-cell proliferation, induced islet vascularization via the VEGF-A/VEGF receptor (VEGFR)-2 signaling pathway, and markedly increased endothelial cell proliferation and microvessel density [131].

Clinical trials have demonstrated that DPP-4i exert beneficial effects in T1D patients. An open-label, parallel-group, randomized controlled trial conducted in 46 adolescents with T1D and diabetic nephropathy recently showed that the use of sitagliptin (administered orally, at a daily dose of 50 mg) as an add-on therapy to the advanced hybrid closed-loop (AHCL) system MiniMed™ 780G (Medtronic, Northridge, CA, USA) was accompanied by a significant decrease in urinary albumin-to-creatinine ratio, 2-h postprandial glucose levels, mean sensor glucose levels, glucose management indicator (GMI) values, coefficient of variation of sensor glucose (a marker of glycemic variability), and total daily dose of insulin, along with a significant increase in time in range (TIR) 3.9-10.0 mmol/L (70–180 mg/dL) and insulin-to-carbohydrate ratio [132]. In this trial, sitagliptin use was safe and well-tolerated, and there were no reported episodes of severe hypoglycemia or diabetic ketoacidosis (DKA) [132]. It is important to note that diabetic nephropathy is associated with an upregulation of DPP-4 expression, suggesting the role of DPP-4 as a potential therapeutic target for the management of this condition [133].

A previously published randomized clinical trial showed that vildagliptin administration (at a daily dose of 50 mg) with iftar meal (together with pre-meal insulin iftar bolus) for the entire month of Ramadan (4 weeks) significantly mitigated postprandial hyperglycemia, increased TIR 70–180 mg/dL, and reduced glycemic variability among Egyptian adolescents and young adults with T1D who were using the MiniMed™ 780G AHCL system (Medtronic, Northridge, CA, USA) [134]. Moreover, there were no reported episodes of severe hypoglycemia or DKA in the study [134].

Another randomized controlled trial conducted in 60 adolescents with T1D and non-alcoholic steatohepatitis (NASH) found that 6-month administration of vildagliptin (administered at a daily dose of 50 mg) as an add-on to insulin therapy improved glycemic control and dyslipidemia, decreased matrix metalloproteinase-14 (MMP-14) levels, and reduced liver stiffness and carotid intima-media thickness (CIMT) [135].

Importantly, the 2-year, two-arm, multicenter, randomized, open-label clinical trial called “PRE1BRAZIL” will investigate the efficacy of the DPP-4i alogliptin (at a dose of 25 mg/day) in delaying the progression of stage 2 T1D (pre-symptomatic T1D with dysglycemia) to stage 3 T1D (clinically symptomatic T1D) in Brazilian patients (aged 18 to 35 years) [136].

## 5. Role of DPP-4i in Solid Organ and Cell Transplantation

The use of DPP-4i has been associated with beneficial clinical outcomes in patients undergoing solid organ and hematopoietic stem cell transplantation.

Ergin et al. [137] conducted a retrospective study on 26 patients who developed noninsulin-dependent hyperglycemia at least one year after pancreas transplantation. Authors showed that the time to insulin requirement was significantly longer among patients (n = 11) who received sitagliptin shortly after the occurrence of hyperglycemia, as compared to patients (n = 15) who did not receive any oral or non-insulin injectable glucose-lowering medications until insulin was clearly required to manage hyperglycemia [137]. These results suggested that early treatment of hyperglycemia can prolong insulin-free graft function after pancreas transplantation. Another retrospective cohort study conducted on 312 patients (of whom 234 with T1D) who underwent pancreas transplantation suggested that post-transplant DPP-4i administration may improve clinical outcomes (including beta-cell function) in this population [138]. Among the 312 study participants, 147 (47%) had received DPP-4i for more than 30 days (DPP-4i group), while 165 (53%) had either not received DPP-4i (n = 137) or had received DPP-4i for less than 30 days (n = 28) (non-DPP-4i group) following pancreas transplantation. Authors documented that patients who used DPP-4i had significantly higher serum C-peptide levels for up to 24 months after transplantation. During the 15-year follow-up period, patients in the DPP-4i group, as compared to those in the non-DPP-4i group, exhibited a significantly higher overall and death-censored pancreas graft survival [138].

In NOD mice, DPP-4 inhibition before and after islet transplantation can reduce the detrimental effects of beta-cell autoimmunity and prolong islet graft survival, partly by reducing the homing of CD4+ T cells to pancreatic beta cells [139]. An uncontrolled, open-label study conducted on 8 islet transplant recipients with evidence of early graft insufficiency showed that 6-month combination therapy with sitagliptin (at a dose of 100 mg/day) plus the proton pump inhibitor pantoprazole (at a dose of 40 mg twice daily) restored insulin independence in one quarter of the study participants (n = 2), although this beneficial effect was not sustained when treatment was withdrawn [140].

In the context of transplantation, DPP-4i may also counteract the detrimental effects of immunosuppressive drugs on pancreatic islets. Indeed, a study conducted in rats found that DPP-4 inhibition alleviated the pancreatic islet dysfunction induced by the immunosuppressant tacrolimus by exerting antioxidant and antiapoptotic actions through the enhancement of GLP-1 receptor signaling [141]. These findings are highly relevant, since post-transplant diabetes mellitus is a common post-transplant complication caused by immunosuppressive drugs [142]. In a 3-month pilot study conducted on 15 kidney transplant recipients who had been diagnosed with new-onset diabetes after transplantation (NODAT) and were treated with sitagliptin (at a standard daily dose of 100 mg, with dosing adjusted according to the estimated glomerular filtration rate) in addition to low-dose tacrolimus (target levels: 2–4 ng/mL) and sirolimus (target levels: 4–6 ng/mL), no significant changes in estimated glomerular filtration rate (eGFR) or in tacrolimus and sirolimus drug levels and doses were observed over the course of the study [143]. Mean HbA1c values significantly decreased from 7.2% (at baseline) to 6.7%, while no patient discontinued sitagliptin due to side effects [143]. Yamada et al. [144] retrospectively reviewed the records of patients who underwent lung transplantation and analyzed data regarding subjects with diabetes mellitus at 6 months post-transplantation. Of 102 patients with diabetes mellitus, 29 were treated with DPP-4i. The 5-year overall survival rates were 77.0% and 44.3%, while the 5-year chronic lung allograft dysfunction (CLAD)-free survival rates were 77.8% and 49.1% in patients who were treated with DPP-4i and in patients who were not treated with DPP-4i, respectively. Moreover, DPP-4/CD26 expression was detected in the CLAD grafts of patients who were not treated with DPP-4i [144]. Similarly, a study conducted on 221 lung transplant recipients documented that CLAD was absent in 34 patients who were treated with sitagliptin (as compared to a CLAD incidence of 18% in subjects who did not use sitagliptin; *p* = 0.02) [145]. The 5-year survival was significantly higher (80% vs. 58%) and the incidence of acute cellular rejection was significantly lower (7% vs. 35%) in patients who used sitagliptin as compared to patients who did not use DPP-4i. Remarkably, immunohistochemical analysis of lung biopsies revealed CD26 expression in perifibrotic areas of CLAD lesions [145].

Furthermore, a growing body of evidence has shown that DPP-4i may also represent a valid therapeutic tool against acute GVHD. In a phase 2, non-randomized clinical trial conducted by Farag et al. [146] on 36 patients, high-dose sitagliptin (administered at a dose of 600 mg every 12 h from the day before transplantation through post-transplant day 14), in combination with sirolimus and tacrolimus, reduced the incidence of grade II–IV acute GVHD by day 100 following myeloablative allogeneic hematopoietic stem cell transplantation. No toxic effects were related to sitagliptin by the investigators [146]. Bacigalupo et al. [147] showed that the anti-CD26 monoclonal antibody begelomab induced over 60% responses among patients with steroid refractory acute GVHD, thereby suggesting that CD26+ T cells may participate in the GVHD-related tissue damage. A prospective, multicenter, open-label, randomized controlled trial conducted on 191 patients receiving alternative donor transplantation showed that sitagliptin (combined with conventional prophylaxis) led to a significant reduction of grade II–IV acute GVHD [148]. Study participants with hematologic malignancies who underwent their first allogeneic hematopoietic stem cell transplantation in first or second complete remission state received busulfan and cyclophosphamide myeloablative conditioning regimen followed by alternative donor transplantation. Sitagliptin was administered at high doses (600 mg every 12 h from day −1 to +14) in addition to a conventional prophylaxis regimen including a calcineurin inhibitor (cyclosporin A/tacrolimus), methotrexate, mycophenolate mofetil, and anti-thymocyte globulin. By day +100, subjects in the sitagliptin group, as compared to subjects in the control group (who received only conventional prophylaxis), exhibited a significantly lower cumulative incidence rate of grade II–IV acute GVHD (15.1% vs. 28.6%, respectively; *p* = 0.019). Overall, the regimen including sitagliptin was well-tolerated, and no significant differences were reported between the sitagliptin group and the control group in terms of Epstein-Barr virus reactivation, cytomegalovirus reactivation, and complications related to the transplant [148]. These findings suggested that DPP-4 inhibition may serve as a potential immunomodulatory strategy for the prophylaxis of acute GVHD.

## 6. Vitamin D and DPP-4i Combination Therapy (VIDPP-4i) in Autoimmune Diabetes and Its Potential as an Adjuvant Treatment Strategy to Improve the Outcomes of Novel Beta-Cell Replacement Therapies

Both clinical and preclinical studies demonstrated that the combined administration of vitamin D and DPP-4i—which we refer to as VIDPP-4i—is associated with more pronounced anti-inflammatory, antioxidant and immunomodulatory effects as compared to the single use of vitamin D or DPP-4i [18,149,150,151], thus suggesting that vitamin D and DPP-4i exert synergistic anti-inflammatory, antioxidant and immunomodulatory actions.

Interestingly, a study showed that VIDPP-4i (based on the co-administration of vitamin D3 and linagliptin) rescued spermatogenesis and testicular steroidogenesis in rats exposed to cisplatin by reducing endoplasmic reticulum stress and activation of nuclear factor kappa B (NF-κB)/inducible nitric oxide synthase (iNOS) [151]. Noteworthy, a recent in silico computational study found that vitamin D3 is also capable of inhibiting DPP-4 [152]. This study also found that combination therapy with the DPP-4 inhibitor vildagliptin (at a daily dose of 10 mg/kg) and vitamin D3 (at a daily dose of 10 µg/kg) considerably elevated serum GLP-1 levels (as compared to vildagliptin alone) in rats with metabolic syndrome [152]. On the other hand, clinical studies conducted in subjects with T2D documented that the use of DPP-4i, as compared to other antidiabetic medications, is associated with a better vitamin D status [153,154]. Interestingly, it has been shown that vitamin D and DPP4i exert synergistic anti-inflammatory actions in patients with T2D by upregulating FOXP3 and interleukin (IL)-37 and by reducing the production of pro-inflammatory cytokines, such as IL-17 and interferon (IFN)-γ [149].

The abovementioned findings may partly explain the beneficial effects (with respect to the preservation of residual beta-cell function) deriving from the use of adjuvant VIDPP-4i combination therapy in individuals with autoimmune diabetes, including T1D and latent autoimmune diabetes in adults (LADA) [18,155,156]. We first documented a substantially prolonged clinical remission phase (4-year clinical remission), decreased glutamic acid decarboxylase antibody (GADA) titers, and preserved beta-cell function in two patients with recent-onset T1D who received VIDPP-4i [sitagliptin (at a daily dose of 100 mg) plus vitamin D3 (at a daily dose of 5000 IU)] in addition to insulin therapy [157]. In a subsequent retrospective study involving 46 patients with recent-onset T1D, co-administration of insulin therapy plus vitamin D3 (at a daily dose of 2000–5000 IU) and sitagliptin (at a daily dose of 50–100 mg), as compared to insulin therapy alone, was associated with a greater frequency and prolonged duration of the clinical remission phase, with 14.8% of patients maintaining insulin independence at 24 months from initiation of VIDPP-4i combination therapy [158]. Similarly, Rapti et al. [159] documented that 2-year combination therapy with metformin (dose: 850 mg twice daily), sitagliptin (dose: 50 mg twice daily), and vitamin D3 (dose: 2000 IU/day) led to normalization of HbA1c values and negativization of GADA in a 31-year-old man with LADA.

A multicenter, randomized, controlled trial investigated the role of 24-month saxagliptin plus vitamin D combination therapy (saxagliptin: 5 mg/day; vitamin D3: 2000 IU/day) as an add-on to conventional therapy in patients with adult-onset autoimmune T1D [155]. As compared to patients who received conventional therapy alone, patients who received saxagliptin plus vitamin D combination therapy—particularly those with higher GADA levels—exhibited a significantly smaller reduction from baseline in the 2-h MMTT C-peptide AUC values [155]. Another multicenter, randomized controlled trial conducted in 60 patients with LADA showed that 12-month co-administration of vitamin D3 and saxagliptin (at daily doses of 2000 IU/day and 5 mg, respectively), in addition to conventional therapy, was associated with stabilization of fasting C-peptide, 2-h postprandial C-peptide and C-peptide index values, and was accompanied by a significant reduction in GADA titers as compared to baseline [156]. In the aforementioned studies [155,156,157,158,159], the co-administration of vitamin D3 and DPP-4i appeared to be safe and well-tolerated. Table 1 summarizes the main clinical studies investigating the role of adjuvant combination therapy with vitamin D and DPP-4i (VIDPP-4i) in patients with autoimmune diabetes (T1D and LADA).

Based on the aforementioned preliminary findings, larger randomized controlled trials are warranted to investigate the long-term efficacy of VIDPP-4i combination therapy in patients with T1D and LADA, as well as its potential role as an adjuvant combination therapy aimed at promoting successful long-term outcomes of novel beta-cell replacement strategies for the treatment of T1D. These trials would also help establish the long-term safety of VIDPP-4i combination therapy in patients with autoimmune diabetes across different age groups, particularly regarding side effects that may result from the concomitant use of DPP-4i and vitamin D (such as upper respiratory tract infections, nasopharyngitis, hypoglycemia, hypercalcemia, and hypercalciuria) [160,161].

Another aspect that needs clarification concerns the most proper doses of vitamin D and DPP-4i that should be investigated in clinical studies on beta-cell replacement therapies for T1D. With regard to vitamin D supplementation, high doses are likely to be more effective than low doses in determining the achievement of serum 25(OH)D levels between 40 and 60 ng/mL, which appear to be associated with more pronounced immunomodulatory and anti-inflammatory effects of vitamin D [162]. These remarks regarding intervention dosage may also apply to DPP-4i. In fact, it is interesting to note that single doses of sitagliptin substantially and dose-dependently inhibit plasma DPP-4 activity, with approximately 80% or greater inhibition of DPP-4 activity occurring at a dose of 50 mg (or greater) over 12 h, and at a dose of 100 mg (or greater) over 24 h [163]. Accordingly, high-dose sitagliptin therapy has been associated with beneficial clinical outcomes in patients who underwent allogeneic hematopoietic stem cell transplantation [146,148]. It has also been shown that addition of sitagliptin to insulin therapy may attenuate the progression of carotid atherosclerosis in patients with T2D in a dose-dependent manner [164]. Moreover, the immunomodulatory and anti-inflammatory properties of DPP-4i could be partly driven by the DPP-4 inhibition-mediated increases in circulating concentrations of GIP and GLP-1. Indeed, a recent study conducted in mice receiving cardiac or islet allotransplantation found that GLP-1 receptor can act as a T cell-negative co-stimulatory molecule, and GLP-1 receptor signaling decreases T lymphocyte graft infiltration, alleviates alloimmune response, and prolongs allograft survival [165]. Other preclinical studies conducted in mice demonstrated that GLP-1 receptor agonists can reduce aberrant immune responses and systemic inflammation by acting on GLP-1 receptor in the central nervous system [166,167]. GLP-1 receptor agonism and GIP receptor agonism can also reduce gut inflammation in mice [168,169]. A systematic review and meta-analysis of randomized controlled trials documented that GLP-1 receptor agonists can lead to significant reductions in biomarkers of oxidative stress and inflammation [170]. Furthermore, preliminary clinical evidence has shown that both GLP-1 receptor agonists and the dual GIP/GLP-1 receptor agonist tirzepatide may effectively improve glycemic control, prevent excess weight gain, and preserve residual beta-cell function in subjects with T1D across different disease stages [171].

**Figure 1:**
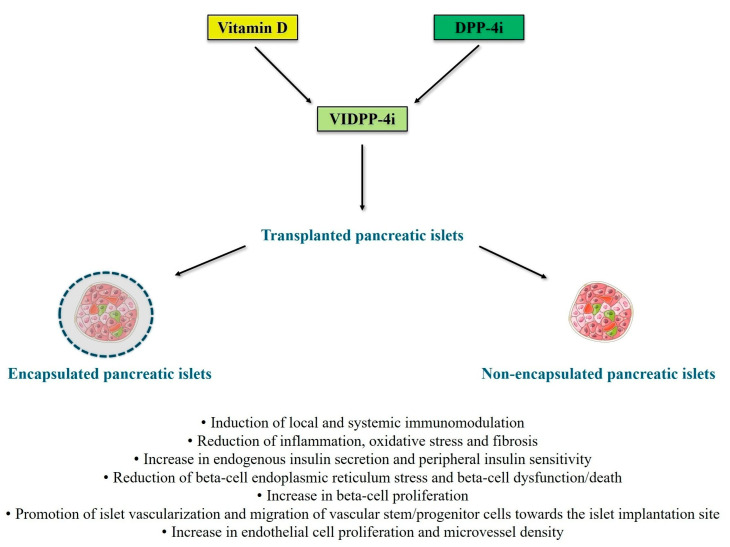
Potential mechanisms of actions through which vitamin D and DPP-4 inhibitor (VIDPP-4i) adjuvant combination therapy may promote successful outcomes of novel beta-cell replacement therapies for type 1 diabetes (particularly long-term islet graft survival and prevention of autoimmunity recurrence and allograft rejection). Figure 1 was created with images adapted from Servier Medical Art licensed under the Creative Commons Attribution 4.0 International License (CC BY 4.0) [URL: https://smart.servier.com/—accessed on 8 July 2025]. The main references for the information presented in the figure are the following: [34,35,36,37,38,39,40,41,42,43,48,49,50,51,56,64,65,66,67,68,79,80,81,82,84,85,88,89,90,91,115,116,117,118,119,120,121,122,125,126,127,128,129,131,151]. Abbreviations: DPP-4i, dipeptidyl peptidase-4 inhibitors; VIDPP-4i, vitamin D and DPP-4 inhibitor combination therapy.

An innovative biomimetic pancreas composed of alpha cells and beta cells differentiated from human induced pluripotent stem cells embedded within a biofunctional matrix with glucose-responsive nanoparticles that encapsulate a GLP-1 analog has recently yielded promising results in C57BL/6 male mice with streptozotocin-induced diabetes mellitus, leading to lower blood glucose levels and higher survival rates of transplanted animals [172]. Thus, novel encapsulation strategies for customized drug delivery may be investigated for the administration of vitamin D [173] and DPP-4i [174] in the context of pancreatic islet transplantation. Indeed, such strategies may maximize the anti-inflammatory, antioxidant, immunomodulatory, and insulinotropic effects of VIDPP-4i within the transplanted pancreatic islets. Figure 1 illustrates the potential mechanisms of actions through which VIDPP-4i combination therapy may promote successful outcomes of novel beta-cell replacement therapies.

## 7. Conclusions

The long-term efficacy of novel beta-cell replacement strategies can be hampered by various factors, such as immune-mediated graft rejection, inadequate vascularization, hypoxia, trauma-induced cell apoptosis, fibrosis, host immune response, and recurrence of autoimmunity. Based on the existing literature in the fields of autoimmune diabetes and solid organ/cell transplantation, the co-administration of vitamin D and DPP-4i (VIDPP-4i adjuvant combination therapy) has the potential to promote and maintain long-term successful outcomes of novel beta-cell replacement therapies for T1D by exerting anti-inflammatory, antioxidant, immunomodulatory, insulinotropic, and insulin-sensitizing effects. In particular, VIDPP-4i may promote the long-term function and survival of transplanted (encapsulated or non-encapsulated) beta cells (including stem cell-derived beta cells) by counteracting beta-cell autoimmunity, favoring immune tolerance, promoting local and systemic immunomodulation, mitigating beta-cell dysfunction/death, and reducing glucotoxicity, lipotoxicity and cytokine-induced toxicity. Additionally, VIDPP-4i may contribute to reduce immunosuppression-related toxicity and to promote proper vascularization of the transplanted islets through the stimulation of angiogenesis and migration of vascular stem/progenitor cells towards the islet transplant site. Therefore, mechanistic studies and randomized controlled trials are warranted to confirm these hypotheses and to define the safety and efficacy profile of VIDPP-4i in T1D patients receiving various types of beta-cell replacement therapies, including those based on the use of encapsulated stem cell-derived beta cells.

## Figures and Tables

**Table 1:** Summary of the main clinical studies investigating the role of adjuvant combination therapy with vitamin D and DPP-4i (VIDPP-4i) in patients with autoimmune diabetes (T1D and LADA).

Study Design	Study Population	Study Treatment	Main Findings
Randomized controlled trial (Yan et al. 2023—Ref. [155])	301 participants with adult-onset autoimmune T1D who were randomly assigned to receive a 24-month conventional therapy alone (metformin with or without insulin), adjunct therapy with saxagliptin, or adjunct therapy with saxagliptin plus vitamin D3.	24-month saxagliptin (5 mg/day) plus vitamin D3 (2000 IU/day) combination therapy [in addition to conventional therapy].	As compared to the conventional therapy alone, 2-h MMTT C-peptide AUC values from baseline to 24 months decreased less with saxagliptin plus vitamin D combination therapy (−276 pmol/L vs. −419 pmol/L; *p* = 0.01) and not to the same extent with saxagliptin therapy alone (−314 pmol/L; *p* = 0.14). For subjects with higher GADA levels, the decline in beta-cell function was significantly lower in saxagliptin plus vitamin D group, as compared to the conventional therapy group. There was a 30% increase in total daily insulin dose in the conventional therapy group (in line with the disease progression), while the increases in total daily insulin dose were only 4.2% in both saxagliptin plus vitamin D group and saxagliptin alone group (*p* = 0.02 and 0.0002, respectively). Overall, saxagliptin and vitamin D were well-tolerated. No adverse events were identified as related to the trial agents.
Randomized controlled trial (Zhang et al. 2020—Ref. [156])	60 patients with LADA were randomized to group A [n = 21; conventional therapy with metformin (daily dose: 1–1.7 g) and/or insulin therapy], group B [n = 20; saxagliptin (daily dose: 5 mg) plus conventional therapy], and group C [n = 19; vitamin D3 (daily dose: 2000 IU/day) plus saxagliptin and conventional therapy] for 12 months.	12-month vitamin D3 (2000 IU/day) plus saxagliptin (5 mg/day) combination therapy [in addition to conventional therapy].	Vitamin D3 plus saxagliptin combination therapy (in addition to conventional therapy) was associated with stabilization of fasting C-peptide levels, 2-h postprandial C-peptide and C-peptide index, and was also accompanied by a significant reduction in GADA titers as compared to baseline. No side effects were reported in any study group.
Retrospective case-control study (Pinheiro et al. 2023—Ref. [158])	46 children, adolescents, and young adults with recent-onset T1D [27 patients with clinical remission at 12 or 24 months served as the case group, and 19 patients without clinical remission at 12 or 24 months served as the control group].	Co-administration of insulin plus sitagliptin (off-label use at a dose of 50–100 mg/day) and vitamin D3 (at a dose of 2000–5000 IU/day).	As compared to insulin therapy alone, co-administration of insulin plus sitagliptin and vitamin D3 was associated with a higher frequency and prolonged duration of the clinical remission phase of T1D. 27 patients were treated with insulin and VIDPP-4i, while 19 patients were treated with insulin alone. Among the 27 patients who used VIDPP-4i, there was a significantly higher prevalence of clinical remission of T1D: 21 patients experienced clinical remission, while 6 patients did not experience clinical remission (*p* = 0.0025). In the VIDPP-4i group, 9 (33.6%) and 4 (14.8%) patients experienced an insulin-free clinical remission (complete clinical remission) at 12 and 24 months, respectively. No severe adverse drug events related to the use of vitamin D3 and sitagliptin combination therapy were reported in the study.
Case reports (Pinheiro et al. 2016—Ref. [157])	A 20-year-old woman and a 21-year-old woman with new-onset T1D.	Sitagliptin (off-label use at a dose of 100 mg/day) and vitamin D3 (5000 IU/day) combination therapy [in addition to insulin therapy].	Sitagliptin and vitamin D3 combination therapy (prescribed in addition to insulin therapy) led to a substantially prolonged clinical remission phase of T1D (4-year clinical remission), which was accompanied by a reduction in GADA titers and preservation of beta-cell function (as demonstrated by fasting C-peptide values at 48 months ≥ 1.0 ng/mL). Both patients maintained normal serum levels of calcium and 25(OH)D. No side effects related to vitamin D3 and sitagliptin combination therapy were reported in the study.
Case report (Rapti et al. 2016—Ref. [159])	A 31-year-old man with LADA and GADA positivity.	Combination therapy with metformin (850 mg twice daily), sitagliptin (50 mg twice daily), and vitamin D3 (2000 IU/day) was prescribed at 3 weeks from the disease diagnosis. The patient refused insulin therapy.	At 2 years from initial disease diagnosis, there were normalization of HbA1c values (5.2% vs. 9.6% at the time of disease diagnosis) and negativization of GADA (without the use of insulin). No side effects related to metformin, sitagliptin, and vitamin D3 combination therapy were reported in the study.

Abbreviations: 25(OH)D, 25-hydroxyvitamin D; AUC, area under the curve; DPP-4i, dipeptidyl peptidase-4 inhibitors; GADA, glutamic acid decarboxylase antibodies; HbA1c, glycated hemoglobin; LADA, latent autoimmune diabetes in adults; MMTT, mixed-meal tolerance test; T1D, type 1 diabetes; VIDPP-4i, vitamin D and DPP-4 inhibitor combination therapy.

## Data Availability

Not applicable.
